# Strong Genetic Influence on a UK Nationwide Test of Educational Achievement at the End of Compulsory Education at Age 16

**DOI:** 10.1371/journal.pone.0080341

**Published:** 2013-12-11

**Authors:** Nicholas G. Shakeshaft, Maciej Trzaskowski, Andrew McMillan, Kaili Rimfeld, Eva Krapohl, Claire M. A. Haworth, Philip S. Dale, Robert Plomin

**Affiliations:** 1 Medical Research Council Social, Genetic and Developmental Psychiatry Centre, Institute of Psychiatry, King's College London, London, United Kingdom; 2 Department of Psychology, University of Warwick, Coventry, United Kingdom; 3 Department of Speech and Hearing Sciences, University of New Mexico, Albuquerque, New Mexico, United States of America; The University of Western Ontario, Canada

## Abstract

We have previously shown that individual differences in educational achievement are highly heritable in the early and middle school years in the UK. The objective of the present study was to investigate whether similarly high heritability is found at the end of compulsory education (age 16) for the UK-wide examination, called the General Certificate of Secondary Education (GCSE). In a national twin sample of 11,117 16-year-olds, heritability was substantial for overall GCSE performance for compulsory core subjects (58%) as well as for each of them individually: English (52%), mathematics (55%) and science (58%). In contrast, the overall effects of shared environment, which includes all family and school influences shared by members of twin pairs growing up in the same family and attending the same school, accounts for about 36% of the variance of mean GCSE scores. The significance of these findings is that individual differences in educational achievement at the end of compulsory education are not primarily an index of the quality of teachers or schools: much more of the variance of GCSE scores can be attributed to genetics than to school or family environment. We suggest a model of education that recognizes the important role of genetics. Rather than a passive model of schooling as instruction (*instruere*, ‘to build in’), we propose an active model of education (*educare*, ‘to bring out’) in which children create their own educational experiences in part on the basis of their genetic propensities, which supports the trend towards personalized learning.

## Introduction

Children differ in their success in learning what is taught at school – skills such as reading and mathematics, and knowledge such as scientific theories and historical facts. To what extent are these individual differences in educational achievement due to nurture or nature? As academic skills and knowledge are taught at school but are seldom explicitly or systematically taught outside of school, it would be reasonable to assume that differences between students in how much they learn are due to differences in how well the educational system teaches these skills and knowledge. From this perspective, it is surprising that quantitative genetic research such as the twin method, which compares identical and fraternal twins, indicates that individual differences in educational achievement are substantially due to genetic differences (heritability) and only modestly due to differences between schools and other environmental differences [1]. For example, we have recently shown in a UK sample of 7,500 pairs of twins assessed longitudinally at ages 7, 9 and 12 that individual differences in literacy and numeracy are significantly and substantially heritable [2]. Across the three ages, the average heritability of literacy and numeracy was 68%, which means that two-thirds of the individual differences (variance) in children's performance on tests of school achievement can be ascribed to genetic differences – i.e., inherited differences in DNA sequence – between them. Remarkably, educational achievement was found to be more heritable than intelligence (68% versus 42%), even though intelligence is not taught directly in schools and is generally viewed as an aptitude of individuals rather than an outcome of schooling.

Although earlier genetic research on school achievement produced a wide range of estimates of heritability, sampling issues may have masked a more consistent pattern. For example, a classic twin study of school achievement found heritabilities of about 40% for English and mathematics in a study of more than 2000 twin pairs [3]. However, heritability estimates in this study are likely to be underestimates due to restriction of range, because the sample was restricted to the highest-achieving high-school twins in the U.S., those who had been nominated by their schools to compete for the National Merit Scholarship Qualifying Test. The wide range of heritability estimates in three other twin studies of general educational achievement is likely to be due to their small sample sizes, which were underpowered to provide reliable point estimates of heritability: Petrill et al., 2010 (314 pairs) [4]; Thompson, Detterman, & Plomin, 1991 (278 pairs) [5]; Wainwright, Wright, Luciano, Geffen, & Martin, 2005 (390 pairs) [6].

In addition to the UK study mentioned above which showed high heritability (68%) for literacy and numeracy (Kovas et al., in press; 7,500 pairs) [2], a study of twins in Australia, the US and Scandinavia has reported high heritability (77%) for reading at age 8 (Byrne et al., 2009; 615 pairs) [7] and in the US at age 10 (Olson et al., 2011; 489 pairs) [8]. Similarly high heritability (62%) has been reported for science performance in 9-year old twins (Haworth et al., 2008; 2602 pairs) [9]. A Dutch study of 12-year-old twins reported a heritability of 60% for a national test of educational achievement (Bartels et al., 2002; 691 pairs) [10]. Another study of general educational achievement in 12-year-old twins in the Netherlands (1,178 pairs) and in the UK (3,102 pairs) did not have zygosity information (Calvin et al., 2012) [11]. However, these studies estimated identical and fraternal twin resemblance from the proportion of same-sex and opposite-sex twins, and this procedure yielded heritability estimates of about 60% in the Dutch sample and 65% in the UK sample.

The purpose of the present study was to investigate the extent to which the remarkably high heritabilities for educational achievement in the UK persist to the end of compulsory education. Unlike many countries such as the US, the UK has a nationwide examination for educational achievement, called the General Certificate of Secondary Education (GCSE), which most pupils complete at the end of compulsory education, typically at age 16. The GCSE provides a valuable test of the hypothesis of strong genetic influence on educational achievement because the GCSE is administered nationwide under standardised conditions. Furthermore, the GCSE is important for individuals, for society, and for government because it is used to make decisions about further education.

On the basis of the evidence from earlier school years – most specifically, in our research on educational achievement in the UK at ages 7, 9 and 12 – we tested the hypothesis that the high heritability of educational achievement persists to the end of compulsory education, as assessed by the GCSE at age 16. Additional support for this hypothesis comes from a recent report extending the analysis of the UK dataset described above [11] to total GCSE scores at age 16 [12]. As in the previous report for this dataset, zygosity information was not available, but estimating identical and fraternal resemblance from the proportion of same-sex and opposite-sex twins suggested substantial genetic influence on GCSE scores [12]. Although heritability was not reported because of the absence of zygosity information, the imputed correlations for identical and fraternal twins suggest a heritability of about 60%. However, a definitive estimate of the heritability of educational achievement can only be made on the basis of evidence from twins with known zygosity, which was achieved by the present study.

## Materials and Methods

### Participants

Twins in the Twins Early Development Study (TEDS) were recruited from birth records of twins born in England and Wales between 1994 and 1996 [13]. Their recruitment and representativeness have been described previously [14]. Children with severe medical problems or whose mothers had severe medical problems during pregnancy were excluded from the analyses. We also excluded children with uncertain or unknown zygosity, and those whose first language was not English. Zygosity was assessed through a parent questionnaire of physical similarity, which has been shown to be over 95% accurate when compared to DNA testing [15]. For cases where zygosity was unclear from this questionnaire, DNA testing was conducted. After exclusions, the total number of individuals for whom GCSE data were obtained at age 16 was 11,117, including 5,474 pairs with data for both co-twins: 2,008 pairs of monozygotic (MZ) twins, 1,730 pairs of same-sex dizygotic (DZ) twins, and 1,736 pairs of opposite-sex DZ twins. Ethical approval was provided by the King's College London ethics committee (reference: 05/Q0706/228), and the parents of the twins provided informed written consent.

### Measures

The UK nationwide examination for educational achievement at the end of compulsory education is called the General Certificate of Secondary Education (GCSE). English, mathematics and science (the latter comprising physics, chemistry and biology, and taught either as a single- or double-weighted course, or as separate courses for each science) are compulsory. Many schools also require English literature and one or more modern foreign languages, among other subjects. GCSEs are typically available in a diverse range of other subjects, including history, geography, information and communications technology (ICT), music, and physical education (PE). Courses usually begin at age 14 (with some slight variations by school and subject), with exams typically being taken at age 16. There is no mandatory number of GCSEs, but students commonly take between 8–10 subjects, and receiving five or more at grades A*–C is typically a requirement for going on to further education.

Shortly after the completion of their GCSEs, each TEDS family was sent results forms by mail, (followed as necessary by telephone reminders). The forms were completed by the twins' parents, and also included results for qualifications other than GCSEs (e.g., ‘Entry Level Certificates’, designed to fall just below GCSE level), which were not analysed in the present study. In order to permit comparable numerical coding across different qualification types, GCSE results were coded from 11 (A*, the highest grade) to 4 (G, the lowest grade). For all analyses, outliers beyond three standard deviations from the mean were removed.

Pupils can select from a wide range of different GCSE subjects, so for many subjects the sample size is too small to analyse. The present study examined the compulsory courses, and several composites generated from the available data for individual subjects. Future papers will examine those individual subjects, including foreign languages, for which sufficient data exist.

Our main general composite was the mean GCSE grade achieved. We also calculated the number of GCSEs passed at grades A*–C, a metric commonly used for university admissions and government policies. These two composites have the advantage of including the results of all GCSE subjects in our dataset, including those taken too rarely to be analysed individually. We also created composites for the compulsory subjects: English mean grade (the mean of all English GCSEs taken; i.e., language and literature, if both were taken), a science mean composite (the mean of whichever science GCSEs were taken), and an overall ‘core subjects’ mean, which is the mean of the compulsory subjects (when all three were taken): the mathematics GCSE, and the English and science composites. In addition, a ‘humanities’ composite was generated, which is the mean of the most commonly taken humanities subjects: history, religious education (RE), media studies, music, art and drama (for those participants who took one or more of these courses); subjects such as geography are omitted, whose course content varies and which are difficult to classify uncontroversially as either humanities or sciences. The composites are detailed further in Table S1 in File S1.

### Analysis

**Figure 1:**
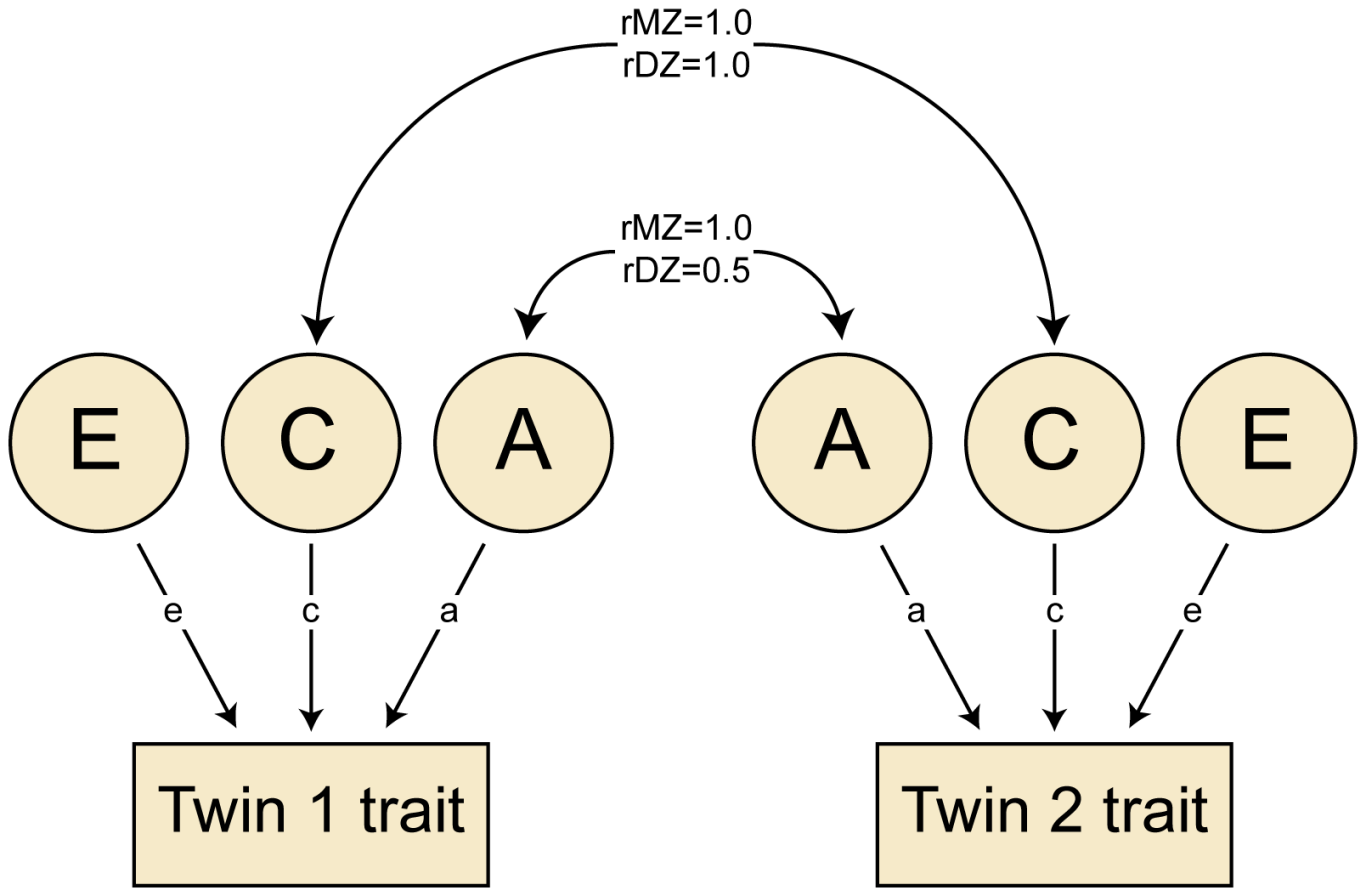
Path diagram representing the basic twin model. A = additive genetic influence; C = shared environmental influence; E = non-shared (unique) environmental influence. Paths a, c and e = effects of A, C and E on the trait. rMZ and rDZ = genetic or shared environmental correlations for monozygotic and dizygotic twins, respectively.

The quantitative genetic model apportions phenotypic variance into additive genetic (A), shared or common environmental (C), and non-shared or unique environmental (E) components [16]. Figure 1 illustrates this ACE model in relation to the twin method. Within MZ twin pairs, both genetic and shared environmental effects by definition correlate 1.0, whereas within DZ twin pairs, shared environmental effects correlate 1.0 but additive genetic effects only correlate 0.5. Non-shared environmental influences are assumed to be uncorrelated for members of a twin pair and thus contribute to differences within pairs. The ACE parameters and their confidence intervals can be estimated by fitting the structural equations implied by the model to the raw data, and decomposing the phenotypic variance/covariance matrices using full-information maximum-likelihood estimation model-fitting (accounting for missing data), as described later. As is standard in twin analyses, residuals correcting for age and sex were used because the age of twins is perfectly correlated across pairs, which would otherwise be misrepresented as shared environmental influence [17]. The same applies to the sex of the twins, since MZ twins are always of the same sex.

Separately for the five twin groups (MZ male pairs and female pairs, same-sex DZ male pairs and female pairs, and opposite-sex DZ pairs), we calculated twin intraclass correlations, which index the proportion of total variance due to between-pair variance [18]. Rough ACE estimates can be calculated from these twin correlations. Heritability, the proportion of phenotypic variance ascribed to heritable genetic influences, can be estimated as twice the difference between the MZ and DZ correlations. Shared environmental influence (environmental influences that make siblings more similar to one another) is the residual familial resemblance not explained by heritability, and can be estimated by subtracting the estimate of heritability from the MZ correlation. The variance that remains is ascribed to non-shared environmental influences specific to each twin within a pair, and measurement error.

When twin correlations are compared by sex as well as zygosity, it is possible to assess quantitative and qualitative sex differences in the genetic and environmental aetiology of individual differences in GCSE scores. Quantitative sex differences refer to differences for ACE parameter estimates for male and female twin pairs. Qualitative sex differences indicate that different genes or different environmental factors influence males and females, which is suggested when the correlation for dizygotic opposite-sex (DZO) twins is less than the correlations for same-sex DZ pairs, based on the assumption that genetic or environmental influences that are specific to one sex will reduce within-pair similarity for the DZO group. It should be noted that regressing out the mean effects of sex from GCSE scores has no bearing on these analyses, which are concerned with the aetiology of variance within the sexes and covariance between the sexes, rather than the phenotypic mean difference between the sexes.

To test the observations derived from the intraclass correlations and to derive ACE estimates and confidence intervals, data for each of the five zygosity-sex groups were analysed in a series of models using the structural equation program OpenMx [19]. These models are based on the standard univariate twin model shown in Figure 1 but extended to a so-called sex-limitation model with the inclusion of DZO twin pairs [20]. Within same-sex twin pairs, the correlation between additive genetic influences on Twin 1 and Twin 2 was fixed at 1.0 for MZ and 0.5 for DZ twin pairs. The correlation between shared environmental influences was fixed at 1.0 for both zygosity groups. Within DZO pairs, in contrast, the genetic and shared environmental correlations may be less than the expected values of 0.5 and 1.0, respectively, if there are significant sex-specific genetic or environmental influences.

The full model allows all parameters to vary across sex: the genetic (or shared environmental) correlation in DZO twins; A, C, and E parameters for boys and girls; and variances for boys and girls. Sex-limitation model fitting involves a series of models that are hierarchically related (nested), which makes it possible to test the relative fit of each alternative model using standard chi-squared difference tests with degrees of freedom equal to the difference in degrees of freedom between the two models [20]. As a test of qualitative sex differences, the fit of the full model was compared to a nested model in which either the genetic or shared environmental correlation was fixed at the expected values of 0.5 and 1.0, respectively (common effects model). As it is not possible to estimate the genetic and shared environmental correlations for DZO twins simultaneously, we cannot ascertain whether any qualitative sex differences are genetic or environmental in origin. As a test of quantitative sex differences, a further nested model (called a scalar model) constrained all ACE parameter estimates to be equal for boys and girls, as well as constraining the genetic correlation to 0.5 in DZO twins; this model is called scalar because it allows differences in phenotypic variance between boys and girls [21]. The third nested model, called the null model, tests for variance differences between boys and girls by constraining all parameters including variances to be equal for males and females. AE, CE and E sub-models within the null model were also tested, fixing the missing ACE parameter(s) to zero in each case. More parsimonious models are typically considered preferable unless a significant deterioration in fit is observed, with ACE estimates being derived from the best-fitting sex-limitation model. Greater detail about sex-limitation modelling in TEDS is available [14]. The model-fitting analyses assume equality of shared environmental effects across MZ and DZ twin pairs, the absence of assortative mating, and independence and additivity of the A, C, and E components [16].

## Results

### Descriptive statistics

Table 1 presents unadjusted raw score means and standard deviations for GCSE scores for the total sample, for all boys and all girls, and for each of the five twin groups. Comparing our results to normative results for GCSE (https://www.gov.uk/government/uploads/system/uploads/attachment_data/file/167426/sfr25-2012.pdf) indicates that our sample is reasonably representative of the UK population: for example, the number of students who receive 5 or more GCSEs with grades of A* to C, an index often used in government policy analyses, is 81.1% nationally and 83.6% in our sample. Mean sex differences can be seen for English, with girls scoring about one-third of a standard deviation higher than boys, and for mathematics, with boys scoring about one-tenth of a standard deviation higher than girls. No significant mean sex differences were found for science. Analysis of variance (ANOVA) was performed on each GCSE score in order to assess the mean effects of sex and zygosity and their interaction. It can be seen from Table 1 that, although significant mean differences emerged for sex and zygosity, they explain less than 3% of the variance. Because GCSE scores are negatively skewed, which is generally interpreted as a ceiling effect, in subsequent analyses, we applied a van der Waerden transformation to all GCSE scores, which normalized the distribution.

**Table 1:** GCSE grade means (standard deviations).

	N	Whole sample	Male	Female	MZm	DZm	MZf	DZf	DZos	Sex	Zyg	Sex×Zyg	R^2^
Mean grade for GCSE passes	11011	8.89 (1.14)	8.77 (1.15)	9.00 (1.12)	8.72 (1.16)	8.83 (1.12)	9.00 (1.12)	8.97 (1.14)	8.90 (1.15)	68.85**	1.47	0.35	0.01
Number of GCSE passes at grade A*–C	11117	8.09 (3.16)	7.81 (3.26)	8.34 (3.04)	7.67 (3.29)	7.96 (3.17)	8.38 (2.97)	8.20 (3.11)	8.12 (3.19)	51.28**	0.90	1.13	<.01
GCSE English mean grade	10928	8.93 (1.17)	8.72 (1.19)	9.11 (1.12)	8.66 (1.20)	8.77 (1.17)	9.10 (1.11)	9.07 (1.14)	8.95 (1.18)	166.47**	4.14*	0.46	0.03
GCSE science mean grade	10166	9.03 (1.25)	9.03 (1.24)	9.03 (1.27)	9.02 (1.23)	9.06 (1.22)	9.03 (1.26)	9.01 (1.29)	9.04 (1.26)	1.77	0.01	0.07	<.01
Mathematics	10852	8.96 (1.40)	9.02 (1.39)	8.90 (1.40)	8.95 (1.41)	9.09 (1.35)	8.91 (1.38)	8.87 (1.42)	8.97 (1.41)	4.75*	1.02	0.29	<.01
GCSE core subjects mean grade	10037	9.05 (1.13)	9.00 (1.13)	9.09 (1.13)	8.96 (1.13)	9.03 (1.11)	9.07 (1.13)	9.07 (1.13)	9.07 (1.14)	15.67**	2.38	0.00	<.01
GCSE humanities mean grade	9349	9.03 (1.33)	8.82 (1.39)	9.20 (1.27)	8.76 (1.39)	8.91 (1.35)	9.19 (1.27)	9.18 (1.30)	9.02 (1.33)	106.51**	1.82	2.16	0.02

* to G. N = sample size after exclusions (individuals); MZ = monozygotic; DZ = dizygotic; m = male; f = female; os = opposite sex. ANOVA performed (on cleaned, normality-transformed data from one randomly-selected twin per pair) to test effects of sex and zygosity: results = F statistic; * = p<.05; ** = p<.01; R^2^ = proportion of variance explained by sex, zygosity and their interaction. All variables except for mathematics are composites. Scores for composite means and mathematics GCSE have a maximum of 11 and a minimum of 4, representing grades A

We also note that the GCSE scores are for the most part highly correlated: .56 on average, excluding subjects with sample sizes too small to analyse individually. Very high correlations were found between English language and English literature (.80), the science subjects (.83 on average), and the ‘core’ subjects of English, science and mathematics (.70 on average); the high phenotypic correlations led us to create composite scores for English, science, the three ‘core’ GCSE subjects (comprising the English and science composites and the mathematics GCSE), and for the overall mean of all subjects. The correlation for the subjects included in the ‘humanities’ composite (history, religious education (RE), media studies, music, art and drama) is somewhat lower on average (.51), but we argue that the traditional division between ‘sciences’ and ‘humanities’ justifies the creation of this composite in order to compare heritability between these areas. Correlation matrices are included in Tables S2 and S3 in File S1, for all subjects with sufficient data, and also for the subset of subjects included in our composites.

For subsequent analyses, the data were age- and sex-regressed as described above.

### Twin correlations

Table 2 presents intraclass twin correlations for all MZ and same-sex DZ twins as well as separately for the five twin groups. Looking first at the twin correlations for all MZ and same-sex DZ twins, the GCSE scores yield MZ correlations that are greater than DZ correlations, suggesting genetic influence. The non-overlapping confidence intervals between the MZ and DZ correlations indicate that the differences are significant. Table 2 includes rough estimates of heritability based on doubling the differences between the MZ and DZ correlations. The average heritability estimate is 53% across the GCSE scores and composites, similar to the mean GCSE score heritability estimate of 52%. Shared environmental influence, estimated as the difference between the MZ correlation and heritability, is 29% on average across the GCSE scores and 36% for the mean GCSE score. A remarkable finding is that the estimates of heritability and shared environmental influence do not differ substantially across diverse subjects. The humanities subjects have the lowest estimate (40%), and science subjects the highest (60%).

**Table 2:** Intraclass twin correlations (with 95% confidence intervals) and approximate variance component estimates for monozygotic (MZ) and same-sex dizygotic (DZss) twins, and separately for MZ and DZ males (m) and females (f), and opposite-sex (os) DZs.

	INTRACLASS CORRELATIONS (95% CONFIDENCE INTERVALS)							VARIANCE COMPONENTS (estimated from MZ vs DZss twin correlations)		
	MZ	DZss	MZm	DZm	MZf	DZf	DZos	h^2^	c^2^	e^2^
Mean grade for GCSE passes	0.88 (0.87–0.89)	0.62 (0.60–0.64)	0.87 (0.85–0.88)	0.57 (0.53–0.62)	0.90 (0.89–0.91)	0.66 (0.63–0.70)	0.55 (0.51–0.58)	0.52	0.36	0.12
Number of GCSE passes at grade A*–C	0.82 (0.81–0.84)	0.57 (0.55–0.60)	0.81 (0.79–0.83)	0.53 (0.48–0.58)	0.83 (0.81–0.85)	0.61 (0.57–0.65)	0.50 (0.47–0.54)	0.50	0.32	0.18
GCSE English mean grade	0.82 (0.80–0.83)	0.56 (0.54–0.58)	0.80 (0.77–0.82)	0.52 (0.47–0.57)	0.83 (0.81–0.85)	0.60 (0.56–0.64)	0.48 (0.44–0.52)	0.52	0.30	0.18
GCSE science mean grade	0.82 (0.80–0.83)	0.52 (0.50–0.55)	0.81 (0.79–0.83)	0.46 (0.40–0.51)	0.83 (0.81–0.85)	0.58 (0.53–0.62)	0.51 (0.47–0.54)	0.60	0.22	0.18
Mathematics	0.82 (0.80–0.83)	0.53 (0.51–0.56)	0.80 (0.78–0.82)	0.50 (0.45–0.55)	0.83 (0.81–0.85)	0.56 (0.51–0.60)	0.45 (0.41–0.49)	0.58	0.24	0.18
GCSE core subjects mean grade	0.87 (0.86–0.88)	0.58 (0.55–0.60)	0.85 (0.83–0.86)	0.53 (0.48–0.58)	0.89 (0.88–0.90)	0.62 (0.58–0.65)	0.53 (0.49–0.56)	0.58	0.29	0.13
GCSE humanities mean grade	0.73 (0.71–0.75)	0.53 (0.50–0.55)	0.71 (0.67–0.74)	0.53 (0.48–0.58)	0.76 (0.73–0.78)	0.52 (0.47–0.57)	0.42 (0.38–0.46)	0.40	0.33	0.27

^2^: double the difference between MZ and DZSS), shared environment (c^2^: the MZ correlation minus h^2^), and unique environment including error (e^2^: 1 - h^2^ - c^2^). All variables except for mathematics are composites. Variance component estimates are heritability (h

The twin correlations are suggestive of sex differences. Looking at the intraclass correlations for the five sex and zygosity twin groups, quantitative sex differences are apparent across most subjects, in that heritabilities are somewhat greater for boys than for girls and shared environmental influences are greater for girls than for boys. There is much less evidence for qualitative sex differences (indicated by lower correlations for opposite-sex DZ twins as compared to same-sex DZ twins), but the correlations are suggestive of such effects for some subjects. These questions are addressed more precisely by the model-fitting results below.

### Model-fitting results

The results seen in the basic twin correlations can be tested more rigorously using model fitting. For all variables, the comparison between nested sex-limitation models described above indicated the presence of significant quantitative sex differences. No qualitative sex differences of any kind were found for any subject.

The finding of quantitative sex differences would suggest that the full sex-limitation model should be used to derive ACE estimates – i.e., separately for males and females. However, the differences between the heritability estimates for males and females are small (e.g., 57% vs. 47%, respectively, for the overall mean GCSE grade), with overlapping confidence intervals for all our measures (see Table S4 in File S1). Despite being statistically significant, therefore, the quantitative sex differences observed are minor, and would probably not be significant for smaller samples (indeed they are not significant for those individual GCSE subjects with small samples in our data). For this reason, the most informative (and parsimonious) model is the null model, with ACE parameter estimates and variances equated between males and females. The AE, CE and E sub-models all resulted in a significant deterioration in fit when compared with the null model, indicating that all the ACE parameters are required. The full sex-limitation model results are available in Table S4 in File S1, together with a comparison of the nested sub-models (Tables S5–11 in File S1).

The null model results are summarised in Table 3; in each case the best-fitting model was an ACE model that included additive genetic effects (A) and shared environmental effects (C), in addition to residual variance (E) not accounted for by A or C.

**Table 3:** Model fitting results for additive genetic (A), shared environmental (C) and residual (E; i.e., non-shared environment and error) components of variance, with 95% confidence intervals.

	Variance components (95% confidence intervals)			Sample (numbers of pairs)				
	A	C	E	MZm	DZm	MZf	DZf	DZos
Mean grade for GCSE passes	0.52 (0.47–0.58)	0.36 (0.31–0.41)	0.11 (0.11–0.12)	891	820	1108	935	1743
Number of GCSE passes at grade A*–C	0.51 (0.45–0.57)	0.32 (0.26–0.37)	0.17 (0.16–0.19)	898	824	1114	940	1759
GCSE English mean grade	0.52 (0.46–0.58)	0.31 (0.24–0.36)	0.18 (0.17–0.19)	881	812	1104	928	1728
GCSE science mean grade	0.58 (0.52–0.66)	0.24 (0.17–0.30)	0.18 (0.16–0.19)	831	770	1018	865	1598
Mathematics	0.55 (0.49–0.62)	0.26 (0.20–0.32)	0.18 (0.17–0.20)	879	799	1085	928	1719
GCSE core subjects mean grade	0.58 (0.52–0.64)	0.29 (0.23–0.35)	0.13 (0.12–0.14)	819	753	1007	856	1573
GCSE humanities mean grade	0.42 (0.35–0.51)	0.32 (0.24–0.39)	0.26 (0.24–0.28)	715	670	974	811	1492

Numbers of pairs are shown for male (m), female (f) and opposite sex (os) monozygotic (MZ) and dizygotic (DZ) twins; figures include incomplete pairs (i.e., those with missing data for one twin). All variables except for mathematics are composites.

These model-fitting results confirm the major conclusions gleaned from the twin correlations. First, heritability is substantial across all GCSE scores. The average heritability is 53%, similar to the heritability of 52% for the mean GCSE score. Second, shared environmental influence is significant for all GCSE scores, but these shared environment estimates are much lower than the heritability. The average shared environment estimate is 30%, and 36% for the mean GCSE score. Third, these estimates do not vary much across most GCSE scores, with heritability estimates for the core subjects all falling into the 52–58% range, and shared environmental variance for these subjects ranging from 24–31%.

One striking finding, closely echoing the estimates derived from the twin correlations in Table 2, is the apparent distinction between the subjects loosely termed as ‘sciences’ or ‘humanities’: the science subjects, on average, are the most heritable (58%), and the humanities the least (42%). The non-overlapping confidence intervals for the heritability estimates suggest that this difference is significant.

## Discussion

Our results indicate that individual differences in educational achievement are just as strong at the end of compulsory education at age 16 as they are in the earlier school years. Heritability is substantial not only for the core subjects of English (52%), mathematics (55%) and science (58%), but also for the (usually optional) humanities subjects in our dataset (42%). We discuss below the implications of finding that GCSE scores are highly heritable.

Also important is the finding that shared environment accounts for much less variance than does genetics. On average, genetics accounts for almost twice as much of the variance of GCSE scores (53%) as does shared environment (30%), even though shared environmental influences include all family, neighbourhood, and school influences that are shared by members of twin pairs growing up together and attending the same school. In addition, estimates of shared environment are also similar across subjects: English (31%), mathematics (26%), science (24%), and the humanities (32%).

Quantitative sex differences emerged for most subjects, with heritability generally greater for boys and shared environmental influence greater for girls (see Table S4 in File S1). Despite the small effect sizes, it is interesting to speculate about how such a pattern of results could occur; for example, girls might be more susceptible to the shared environmental influences of schools or peers. However, we prefer merely to note these significant sex differences in our sample and to defer speculation about their origins until these results are replicated, for reasons discussed later.

We discuss each of these three topics, acknowledge limitations of our study, and conclude by discussing the policy implications of finding such strong genetic influence and moderate shared environmental influences on educational achievement at the end of compulsory education.

### Why is there such strong genetic influence for all GCSE subjects?

It was surprising to us to find such strong genetic influence on educational achievement in the early school years, and now, as seen in the present results, at the end of the compulsory school years as well. The surprise stems from thinking that, as these subjects are taught at school, differences in educational achievement are primarily due to differences in teaching. This thinking is not entirely wrong-headed: differences between schools account for about a third of the variance in educational achievement [22]. However, most of the variance in achievement lies within schools: that is, children within a school differ widely in their performance. Teachers within a school account for some variance, but children in the same classroom also differ widely in their achievement [14]. Neighbourhoods within a school district account for perhaps 10–15% of the variance, but at least half of this variance can be attributed to differences between families [12].

Differences between families could be due to nature or nurture, but the present results indicate that familial resemblance for educational achievement is primarily due to nature rather than nurture. Paradoxically, individual differences in educational achievement may be highly heritable precisely because these subjects are taught at school. To the extent that children receive the same education, which is the goal of a one-size-fits-all national curriculum, this potential source of environmental differences between children's educational achievement is attenuated. As a result, the individual differences that remain will be due to genetic differences to a greater extent. This line of thinking leads to what may be an uncomfortable realisation: success in achieving widely accepted educational goals such as educational equity, social mobility, and personalised learning will all increase heritability. Indeed, heritability could be viewed as an index of equity in educational opportunities.

For this reason, one might predict that countries with a tightly prescribed national curriculum, such as the UK, might yield higher heritability estimates than countries with decentralized educational systems, such as the US. Although cross-country comparisons of twin results have reported such differences, the studies were too small to provide adequate tests of cross-country differences in heritability [7] [23]. One argument against this environmental explanation for the high heritability of educational achievement is that it seems odd, perhaps, that the effect of universal education would emerge full blown in the earliest school years [14]. It also seems odd that the effect does not diminish during the school years as education moves beyond teaching basic skills such as literacy and numeracy. For example, after children learn to read, they read to learn, which might weaken the impact of universal education as children educate themselves to a greater extent; this could be seen as an example of a gene-environment correlation (discussed below), which would have the effect of increasing the heritability estimate beyond the level produced by genes alone.

Another possibility is that educational achievement shows strong genetic influence because it taps into many genetically influenced traits, not just aptitudes of cognition but also appetites of personality and motivation which also have genetic influences. Multivariate genetic analysis, which addresses the genetic and environmental origins of covariance among traits [16], can be used to investigate why educational achievement is so heritable, by identifying the genetic correlates of educational achievement. In other words, multivariate genetic analysis can be used to investigate the extent to which the high heritability of educational achievement is due to the genetic influence of traits such as cognitive abilities, personality, motivation, and adjustment. It can also be used to examine two additional features of the present results: all GCSE scores intercorrelate substantially, 0.56 on average, and all GCSE scores are substantially heritable, 0.53 on average. Although these two findings might suggest that some common genetic mechanisms affect all GCSE scores, it is also possible that each GCSE score could be heritable for different genetic reasons. Multivariate genetic analysis can estimate the extent to which the same genes affect different GCSE scores. Such analyses into genetic correlates of GCSE scores, and genetic intercorrelations among GCSE scores, are the focus of our ongoing analyses, which will be presented in a future paper.

We noted that one possible exception to the finding that all GCSE subjects show strong genetic influence is that subjects loosely termed as ‘sciences’ are more heritable (58%) than ‘humanities’ (42%). This finding is interesting because it is contrary to the ‘folk psychology’ view that science is something you learn from teaching (i.e., environment) but abilities in the humanities are ‘gifts’ (i.e., genetics). Multivariate genetic analyses might help to explain this heritability difference if different patterns of genetic correlates are found for sciences and humanities.

### Why is shared environmental influence so modest for all GCSE subjects?

Just as important as the finding of high heritability is the finding that shared (as opposed to non-shared) environmental influence accounts for 30% of the variance of GCSE scores on average, compared to the 53% accounted for by genetics. On the one hand, it is interesting that so much of the variance is due to shared environment because it often has negligible influence on behavioural traits [24]. This estimate of 30% of the variance of GCSE scores being due to shared environment is greater than what we have found at earlier ages, where the average estimate of shared environmental influence for National Curriculum scores for literacy and numeracy across ages 7, 9 and 10 is 12% [14]. It would be interesting if this jump in shared environmental influence at the end of secondary school proved to be replicable, as it would suggest that secondary schools have more of an impact than primary schools. We are currently obtaining data on school quality to test the hypothesis that the quality of secondary schools mediates this effect.

On the other hand, it is remarkable that only 30% of the variance is due to shared environment for GCSE scores because familial resemblance is indexed in our study using siblings who have grown up in the same family, lived in the same neighbourhood, attended the same school, and perhaps even studied and revised together during their education. In comparison, resemblance between parents and offspring is more limited environmentally because parents and offspring grow up at least two decades apart, and in different homes; their resemblance is also limited genetically because different genes can affect adults (parents) and children (offspring). Moreover, the siblings in our study are twins, which means that they also lived together prenatally in the same womb and grew up together at exactly the same age. In other words, twin siblings maximally share their environments, and yet our results indicate that their resemblance owes substantially more to genetics than to shared environment.

It should be mentioned that even this modest estimate of shared environmental influence might be inflated. Twins have been reported to have twin-specific shared environmental effects – that is, environmental effects that are shared by twins but not by other siblings – such as the extra resemblance that might be derived from growing up together at exactly the same age [25]. Data from the recent sibling study of GCSE scores [12] appear to provide at most modest support for this hypothesis, because correlations for DZ twins are only slightly greater than correlations for non-twin siblings: the GCSE correlation for DZ brothers was 0.62, as compared to 0.59 for non-twin brothers; for DZ sisters and non-twin sisters, the correlations were 0.64 and 0.62, respectively. However, the study did not assess zygosity, so the same-sex DZ correlations may not be accurate.

It should also be noted that the term ‘shared environment’ is shorthand for ‘shared environmental effects’, not ‘shared environmental events’. That is, twins manifestly share environmental events such as the same parents, the same home, and the same school. However, quantitative genetic analyses such as the twin method address the genetic and environmental sources of individual differences, that is, genetic and environmental factors that make a difference. In the case of shared environment, this refers to the influence of environmental factors that contribute to the covariance of siblings after controlling for the genetic contribution to their covariance. In other words, shared environments such as shared families and schools might not have shared environmental effects.

Does finding only modest shared environmental influence mean that schools do not matter? Of course not: schools systematically teach children basic skills such as reading, writing and arithmetic, and basic cultural knowledge. Although the difference in educational achievement between the best schools and the worst schools might not be great compared to the wide range of individual differences within schools, the difference between going to school and not going to school would be enormous. Moreover, shared environmental influence refers to only one specific type of environmental influence: for example, the extent to which children attending the same school are similar in their educational achievement after controlling for genetic influence. Controlling for genetic influence is important: differences between schools cannot be safely assumed to be entirely environmental in origin, because families are not assigned randomly to schools. Genetic factors are likely to contribute to this non-random assortment of children to schools – including the parents' own educational achievement, as discussed later.

Some of the clearest evidence for the impact of schools on intelligence and cognitive development comes from studies which have used the school cut-off method [26]. Children who have just missed the cut-off date for entering school are compared at later times with those who just made the cut-off. The groups are nearly identical in age and many other characteristics, but differ by one year's schooling. Not only does the additional year of schooling have a significant effect on IQ and a range of cognitive tasks, a year of schooling generally has at least twice as much of an effect as does a year of additional age without an additional year of schooling. Thus schooling has a very substantial mean impact, but – based on studies such as the present one – relatively little impact on the relative differences between children.

Environmental effects that are not shared by family members are called non-shared environmental influences [24]. While non-shared environment accounts for only a modest proportion of variance in our sample (very modest, considering that measurement error is included in this estimate), it is still significant. One direction for research is to attempt to identify these non-shared environmental influences on educational achievement. What environmental factors could be responsible for making children in the same classroom in the same school differ so much in their educational achievement? For example, are teachers differentially effective in teaching some children more than others? The difficulty in investigating non-shared environmental influences is to disentangle them from genetic influences. That is, teachers might respond differently to some children on the basis of the children's genetically driven differences. Identical twins are a powerful tool for studying non-shared environment while controlling for genetics. Since members of identical twin pairs are identical in terms of inherited DNA sequences, differences within pairs of identical twins can only be due to non-shared environmental influences. Nonetheless, in general it has proven difficult to identify specific factors that account for non-shared environment [24]. However, some positive results were found in a study of non-shared classroom experiences of MZ twins who were in the same classrooms and were assessed every school day for two weeks. MZ twins experienced their teachers, classrooms, and peers somewhat differently, and these experiential differences within MZ twin pairs were significantly associated with differences in educational achievement, especially in mathematics and science [27]. In relation to our finding that science subjects may be more heritable than humanities subjects, it is interesting that we find *less* non-shared environmental influence for sciences than humanities. Since estimates of non-shared environmental effects include measurement error, one possibility is that humanities are less reliably measured than sciences.

### Sex differences?

When examining the phenotypic variance difference between sexes, we found that individual differences within sex are far greater than average differences between boys and girls. An important point is that the description and causes of individual differences are not necessarily related to the description and causes of average differences between groups. That is, regardless of whether there are mean sex differences, sex differences at the level of individual differences can still be found. Genetic analyses focus on the origins of individual differences for boys and girls, not mean differences. Therefore, the mean differences were regressed prior to model fitting analyses.

For several of our measures, we found significant quantitative (but no qualitative) sex differences: greater heritability for boys, and greater shared environment for girls. However, these differences were small for all measures, with overlapping confidence intervals (Table S4 in File S1). Moreover, we had not anticipated these findings because our research on the same sample in the earlier school years did not find significant quantitative sex differences. For example, at ages 7, 9 and 10, we found similar estimates of heritability and shared environment for boys and girls [14]. Indeed, when quantitative sex differences were found, they were in the opposite direction from those in the present study: heritability was slightly lower for boys, and shared environment slightly lower for girls. It is noteworthy that our finding of quantitative sex differences cannot be tested by comparing correlations for non-twin siblings, because sibling studies cannot separate genetic and environmental influences. If heritability is greater for boys and shared environment is greater for girls, these quantitative sex differences would be counterbalanced; that is, heritability would contribute to a higher correlation for brothers and shared environment would contribute to a higher correlation for sisters. Although our results suggest that the magnitude of these counterbalancing effects is similar – heritability is about 10% greater for boys and shared environment is about 10% greater for girls – in fact, we find a slightly lower average correlation for DZ boys (0.52) than for DZ girls (0.59). In this context, it is noteworthy that in the recent paper on GCSE scores mentioned in the Introduction [12], correlations for non-twin siblings were in a similar direction although the difference was even smaller: 0.59 for brothers and 0.62 for sisters.

For these reasons, although there is some support in the literature for our findings of quantitative sex differences, we suggest caution in accepting and interpreting these results until they are replicated in independent studies.

### Limitations

Limitations of the present study include general limitations of the twin method, most notably the equal environments assumption – that environmentally-caused similarity is equal for MZ and DZ twins – and the assumption that results for twins generalize to non-twin populations [16]. The equal environments assumption has survived several tests of its validity, but the most persuasive evidence is that similar results are found using two other methods with different assumptions: the adoption method and a quantitative genetic method based on DNA alone [28] [29]. In terms of the generalization from twin to non-twin samples, GCSE scores for twins and non-twin siblings have been shown to be very similar in means and variances [12].

Specific limitations involve aspects of the sample and measures. As mentioned earlier, although our sample was relatively large, the sex differences that emerged from our sex-limitation model fitting were so small that caution is warranted in interpreting these results until they are replicated in other studies. In terms of the measure, although the GCSE may not be the best or most thorough test of educational achievement, it is important because it is a nationwide test that is used to make decisions about further education and employment. Moreover, our results for the GCSE at age 16 are comparable to those we obtained using web-based tests of reading and mathematics at age 12 [30]. Our sample tended to score more highly than the national average, and our dataset does not contain information about failed exams (i.e., below grade G), but these account for only around 1.5% of exams nationally (https://docs.google.com/spreadsheet/pub?key=0AoEZjwuqFS2PdEZfSVpFd0UwdExROXlQbHR4d2laUHc). A possible specific limitation of our study is that GCSE scores were reported by parents. However, for 7,367 of the twins, we were able to obtain official GCSE scores from the UK National Pupil Database (http://www.education.gov.uk/researchandstatistics/national-pupil-database); the correlations between parent-reported scores and official scores were 0.98 for English, 0.99 for mathematics, and >0.95 for all science subjects, so obtaining GCSE results from parents was not problematic. Another limitation is that the present analyses are univariate; as mentioned earlier, multivariate genetic analyses are in progress that address the genetic and environmental origins of the phenotypic correlations among GCSE subjects, and those between GCSE scores and other traits.

### A genetic model of education

Education has been slow to take on board the importance of genetics for educational achievement [31] [32] [33]. Some of this reluctance comes from general misconceptions of what it means to say that genetics influences educational achievement. One major misconception is that finding genetic influence diminishes the importance of schools: even if the heritability of educational achievement were 100%, this means that the differences in achievement between pupils are due to genetic differences between them but it would not mean that schools are unimportant. As noted earlier in relation to the modest impact of schools on shared environmental influence, the differential impact of good and bad schools is not great, but the difference between schools and no schools is likely to be enormous. Without educational curricula, whether taught in schools or homes, children would not systematically learn basic skills such as literacy and numeracy or basic knowledge such as history and science. In addition, there is a more subtle way in which schools could be important even if heritability were 100%: heritability of 100% means that inequalities of educational opportunity do not exist. In this counter-intuitive sense, heritability can be considered as an index of equality.

Rather than a universal, one-size-fits-all approach to educational curricula, a more individually tailored approach is needed that recognizes the strong genetic contribution to individual differences in educational achievement. Education is not imposed on a passive organism. When a universal educational curriculum is imposed on children, children differ in their response to it, in large part for genetic reasons [1]. In quantitative genetics, this process is known as genotype-environment interaction, in which the effect of an imposed environment differs as a function of individuals' genetic propensities. However, a farther-reaching view of the interface between the environment and genes is genotype-environment correlation, which denotes genetic influence on exposure to environments. Genotype-environment correlation involves choice of environments rather than the imposition of an environment: children select, modify and create environments in part for genetic reasons [34]. There are three types of genotype-environment correlation: passive, evocative, and active. The passive type occurs because children passively receive environments correlated with their genotypes when they are reared by their genetic parents. For example, parents whose genetic propensities lead them to read more are also likely to read more to their children. Evocative genotype-environment correlation occurs when children, on the basis of their genetic propensities, evoke reactions from other people, such as teachers noticing a child who loves to read and then encouraging that propensity. Active genotype-environment correlation occurs when children select, modify, and construct or re-construct experiences that are correlated with their genetic propensities. For example, children who like to read can cultivate their own reading in the library, on the internet, and via friends.

The passive type of genotype-environment correlation is one reason why it is unsafe to assume that correlations between family background and educational achievement are mediated environmentally. The evocative type occurs to the extent that parents and teachers recognize and foster genetically driven aptitudes and appetites among children. Active genotype-environment correlation has the broadest ramifications for education because it suggests an active model of education in which children actively select, modify and create their own environments, even within an ostensibly ‘universal’ curriculum. Using reading again as an example, children with reading problems will benefit from increased reading instruction but because reading is difficult they are less likely to be motivated to read on their own.

Active genotype-environment correlation may be the most general process by which genotypes develop into phenotypes, in education as well as other developmental domains. The distinction between the prevailing passive model of imposed environments and this active model of education can be captured by the contrast between the word ‘instruction’, which is derived from the Latin word *instruere* meaning ‘to build in’, and the word ‘education’, which is derived from *educare* meaning ‘to bring out’. The instruction model of imposed environments is consistent with a one-size-fits-all national curriculum approach, whereas the education model of active experiences fits the trend towards adaptive learning systems tailored to each pupil [35]. For example, there is increasing evidence that individualized reading instruction is more effective than instruction of similar quality that is not individualized [36]. Genetics will become more specifically useful in such personalized learning programs as specific genes responsible for the high heritability of educational achievement are identified, and the dynamic interplay of genetic and environmental factors, e.g., genotype-environment correlation, is better understood. However, as is the case for complex traits in all of the life sciences, progress has been slow in identifying genes responsible for heritability [37].

In closing, we note that accepting the evidence for strong genetic influence on individual differences in educational achievement has no necessary implications for educational policy, because policy depends on values as well as knowledge. For example, a deep-seated fear is that accepting the importance of genetics justifies inequities – educating the best and forgetting the rest. However, depending on one's values, the opposite position could be taken, such as putting more educational resources into the lower end of the distribution to guarantee that all children reach minimal standards of literacy and numeracy, so that they are not excluded from our increasingly technological societies. It is to be hoped that better policy decisions will be made with knowledge than without. Part of that knowledge is the strong genetic contribution to individual differences in educational achievement.

## Supporting Information

File S1 **Supporting information tables. Table S1.** Construction of composites. **Table S2.** Correlation matrix for all GCSE subjects. **Table S3.** Correlation matrix for subjects included in composites. **Table S4.** Sex limitation A, C and E estimates. **Table S5.** Sex limitation sub-model comparisons: Mean grade for GCSE passes. **Table S6.** Sex limitation sub-model comparisons: Number of GCSE passes at grade A*–C. **Table S7.** Sex limitation sub-model comparisons: GCSE English mean grade. **Table S8.** Sex limitation sub-model comparisons: GCSE science mean grade. **Table S9.** Sex limitation sub-model comparisons: Mathematics. **Table S10.** Sex limitation sub-model comparisons: GCSE core subjects mean grade. **Table S11.** Sex limitation sub-model comparisons: GCSE humanities mean grade.
(PDF)
