# Supplementary material for: Strong Genetic Influence on a UK Nationwide Test of Educational Achievement at the End of Compulsory Education at Age 16
Source: PLoS One. 2013 Dec 11;8(12):e80341. doi: 10.1371/journal.pone.0080341 (PMC3859476; doi:10.1371/journal.pone.0080341)
Supplement: File S1 — Supporting information tables. Table S1. Construction of composites. Table S2. Correlation matrix for all GCSE subjects. Table S3. Correlation matrix for subjects included in composites. Table S4. Sex limitation A, C and E estimates. Table S5. Sex limitation sub-model comparisons: Mean grade for GCSE passes. Table S6. Sex limitation sub-model comparisons: Number of GCSE passes at grade A*–C. Table S7. Sex limitation sub-model comparisons: GCSE English mean grade. Table S8. Sex limitation sub-model comparisons: GCSE science mean grade. Table S9. Sex limitation sub-model comparisons: Mathematics. Table S10. Sex limitation sub-model comparisons: GCSE core subjects mean grade. Table S11. Sex limitation sub-model comparisons: GCSE humanities mean grade. (PDF) [file pone.0080341.s001.pdf]

Supplementary Online Material

Contents:

- Table S1: Details of construction for each subject / composite assessed.
- Table S2: Correlation matrix (Pearson's *r*) for all GCSE subjects in our dataset, excluding short-course GCSEs and subjects with sample sizes too small to analyse individually. Significance (2-tailed) and sample size (N) is shown for each.
- Table S3: Correlation matrix (Pearson's *r*) for GCSE subjects included in composites.
- Table S4: Sex limitation model fitting results (with 95% confidence intervals), showing A, C and E estimates separately for males and females.
- Tables S5-S11: Sex limitation sub-model comparisons.

Table S1: Construction of composites.

Mean grade for GCSE passes	All GCSE subjects in dataset, including those with sample sizes too small to analyse individually.
Number of GCSE passes at grade A*-C	All GCSE subjects in dataset, including those with sample sizes too small to analyse individually.
GCSE English mean grade	Mean of: English language, English literature (whichever taken)
GCSE science mean grade	Mean of: science core, science additional, biology, chemistry, physics (whichever taken)
Mathematics	Single GCSE; raw grade used
GCSE core subjects mean grade	Mean of: English composite, mathematics grade, science composite (requiring all three)
GCSE humanities mean grade	Mean of: media studies, history, religious education (RE), art, drama and music (whichever taken)

Table S2: Correlation matrix for all GCSE subjects (continues on next page).

		English Language	English Literature	Media Studies	Mathematics	Statistics	Science Core	Science Additional	Biology	Chemistry	Physics	History	Geography	RE	French	German	Spanish	DT	ICT	Business Studies	Art and Design	PE	Drama	Music
English Language	Correlation	1	.800	.609	.691	.646	.659	.631	.646	.633	.631	.729	.733	.683	.666	.634	.673	.611	.496	.609	.551	.546	.563	.526
	Sig. (2-tailed)		.000	.000	.000	.000	.000	.000	.000	.000	.000	.000	.000	.000	.000	.000	.000	.000	.000	.000	.000	.000	.000	.000
	N	5466	4791	461	5398	627	2890	2349	2159	2147	2142	2369	2082	2566	1884	780	731	2078	1166	775	1607	1325	692	562
English Literature	Correlation	.800	1	.549	.618	.597	.601	.586	.601	.599	.595	.696	.697	.638	.633	.619	.621	.599	.478	.601	.520	.500	.549	.535
	Sig. (2-tailed)	.000		.000	.000	.000	.000	.000	.000	.000	.000	.000	.000	.000	.000	.000	.000	.000	.000	.000	.000	.000	.000	.000
	N	4791	4832	366	4775	592	2513	2109	2065	2053	2047	2239	1933	2335	1818	754	689	1826	1034	711	1436	1162	631	541
Media Studies	Correlation	.609	.549	1	.508	.489	.498	.520	.539	.429	.420	.591	.649	.434	.562	.559	.382	.532	.390	.537	.439	.338	.621	.674
	Sig. (2-tailed)	.000	.000		.000	.000	.000	.000	.000	.000	.000	.000	.000	.000	.000	.000	.011	.000	.000	.000	.000	.001	.000	.000
	N	461	366	467	455	57	298	239	125	124	124	145	120	188	109	57	44	150	104	71	128	96	73	33
Mathematics	Correlation	.691	.618	.508	1	.785	.752	.735	.734	.763	.775	.686	.721	.595	.643	.611	.670	.606	.545	.602	.483	.577	.423	.536
	Sig. (2-tailed)	.000	.000	.000		.000	.000	.000	.000	.000	.000	.000	.000	.000	.000	.000	.000	.000	.000	.000	.000	.000	.000	.000
	N	5398	4775	455	5461	627	2889	2347	2157	2145	2139	2363	2080	2559	1878	783	727	2081	1162	773	1606	1320	691	567
Statistics	Correlation	.646	.597	.489	.785	1	.675	.661	.700	.669	.688	.625	.653	.614	.611	.597	.630	.605	.580	.600	.447	.425	.133	.548
	Sig. (2-tailed)	.000	.000	.000	.000		.000	.000	.000	.000	.000	.000	.000	.000	.000	.000	.000	.000	.000	.000	.000	.000	.335	.000
	N	627	592	57	627	629	276	247	333	334	333	269	257	290	236	115	90	232	168	104	171	157	55	75
Science Core	Correlation	.659	.601	.498	.752	.675	1	.831				.664	.720	.594	.612	.540	.613	.529	.460	.629	.422	.534	.439	.468
	Sig. (2-tailed)	.000	.000	.000	.000	.000		.000				.000	.000	.000	.000	.000	.000	.000	.000	.000	.000	.000	.000	.000
	N	2890	2513	298	2889	276	2931	2372				1199	1025	1369	855	337	345	1165	708	444	908	789	421	280
Science Additional	Correlation	.631	.586	.520	.735	.661	.831	1				.650	.699	.568	.651	.600	.622	.550	.444	.550	.449	.523	.428	.505
	Sig. (2-tailed)	.000	.000	.000	.000	.000	.000					.000	.000	.000	.000	.000	.000	.000	.000	.000	.000	.000	.000	.000
	N	2349	2109	239	2347	247	2372	2372				1018	883	1145	769	296	305	960	557	368	733	620	335	243
Biology	Correlation	.646	.601	.539	.734	.700			1	.828	.821	.678	.707	.620	.610	.574	.633	.535	.491	.646	.463	.530	.368	.399
	Sig. (2-tailed)	.000	.000	.000	.000	.000				.000	.000	.000	.000	.000	.000	.000	.000	.000	.000	.000	.000	.000	.000	.000
	N	2159	2065	125	2157	333			2174	2145	2137	1093	987	1046	989	427	368	773	426	300	598	470	228	270
Chemistry	Correlation	.633	.599	.429	.763	.669			.828	1	.834	.681	.690	.609	.637	.544	.616	.548	.479	.612	.471	.541	.321	.402
	Sig. (2-tailed)	.000	.000	.000	.000	.000			.000		.000	.000	.000	.000	.000	.000	.000	.000	.000	.000	.000	.000	.000	.000
	N	2147	2053	124	2145	334			2145	2162	2141	1091	985	1032	983	429	365	766	425	298	590	467	221	269
Physics	Correlation	.631	.595	.420	.775	.688			.821	.834	1	.673	.651	.584	.615	.572	.583	.519	.469	.625	.448	.558	.354	.443
	Sig. (2-tailed)	.000	.000	.000	.000	.000			.000	.000		.000	.000	.000	.000	.000	.000	.000	.000	.000	.000	.000	.000	.000
	N	2142	2047	124	2139	333			2137	2141	2157	1081	981	1035	977	428	366	766	423	294	585	467	226	269
History	Correlation	.729	.696	.591	.686	.625	.664	.650	.678	.681	.673	1	.745	.689	.643	.590	.620	.610	.504	.670	.505	.577	.410	.483
	Sig. (2-tailed)	.000	.000	.000	.000	.000	.000	.000	.000	.000	.000		.000	.000	.000	.000	.000	.000	.000	.000	.000	.000	.000	.000
	N	2369	2239	145	2363	269	1199	1018	1093	1091	1081	2388	828	1179	967	406	350	766	467	278	614	504	289	243
Geography	Correlation	.733	.697	.649	.721	.653	.720	.699	.707	.690	.651	.745	1	.678	.653	.606	.653	.673	.515	.658	.575	.639	.558	.517
	Sig. (2-tailed)	.000	.000	.000	.000	.000	.000	.000	.000	.000	.000	.000		.000	.000	.000	.000	.000	.000	.000	.000	.000	.000	.000
	N	2082	1933	120	2080	257	1025	883	987	985	981	828	2100	953	838	325	299	734	412	254	551	517	195	190

		English Language	English Literature	Media Studies	Mathematics	Statistics	Science Core	Science Additional	Biology	Chemistry	Physics	History	Geography	RE	French	German	Spanish	DT	ICT	Business Studies	Art and Design	PE	Drama	Music
RE	Correlation	.683	.638	.434	.595	.614	.594	.568	.620	.609	.584	.689	.678	1	.584	.520	.591	.591	.480	.558	.491	.543	.531	.555
	Sig. (2-tailed)	.000	.000	.000	.000	.000	.000	.000	.000	.000	.000	.000	.000		.000	.000	.000	.000	.000	.000	.000	.000	.000	.000
	N	2566	2335	188	2559	290	1369	1145	1046	1032	1035	1179	953	2587	930	377	352	992	599	369	751	619	345	273
French	Correlation	.666	.633	.562	.643	.611	.612	.651	.610	.637	.615	.643	.653	.584	1	.799	.776	.539	.479	.607	.524	.568	.401	.489
	Sig. (2-tailed)	.000	.000	.000	.000	.000	.000	.000	.000	.000	.000	.000	.000	.000		.000	.000	.000	.000	.000	.000	.000	.000	.000
	N	1884	1818	109	1878	236	855	769	989	983	977	967	838	930	1896	143	202	637	360	240	535	433	241	241
German	Correlation	.634	.619	.559	.611	.597	.540	.600	.574	.544	.572	.590	.606	.520	.799	1	.867	.504	.450	.579	.405	.448	.400	.440
	Sig. (2-tailed)	.000	.000	.000	.000	.000	.000	.000	.000	.000	.000	.000	.000	.000	.000		.000	.000	.000	.000	.000	.000	.000	.000
	N	780	754	57	783	115	337	296	427	429	428	406	325	377	143	787	45	286	162	104	238	164	85	93
Spanish	Correlation	.673	.621	.382	.670	.630	.613	.622	.633	.616	.583	.620	.653	.591	.776	.867	1	.479	.343	.500	.473	.474	.538	.556
	Sig. (2-tailed)	.000	.000	.011	.000	.000	.000	.000	.000	.000	.000	.000	.000	.000	.000	.000		.000	.000	.000	.000	.000	.000	.000
	N	731	689	44	727	90	345	305	368	365	366	350	299	352	202	45	735	224	145	102	191	152	88	78
DT	Correlation	.611	.599	.532	.606	.605	.529	.550	.535	.548	.519	.610	.673	.591	.539	.504	.479	1	.469	.566	.525	.487	.336	.335
	Sig. (2-tailed)	.000	.000	.000	.000	.000	.000	.000	.000	.000	.000	.000	.000	.000	.000	.000	.000		.000	.000	.000	.000	.000	.000
	N	2078	1826	150	2081	232	1165	960	773	766	766	766	734	992	637	286	224	2102	482	293	603	494	194	166
ICT	Correlation	.496	.478	.390	.545	.580	.460	.444	.491	.479	.469	.504	.515	.480	.479	.450	.343	.469	1	.452	.363	.316	.428	.415
	Sig. (2-tailed)	.000	.000	.000	.000	.000	.000	.000	.000	.000	.000	.000	.000	.000	.000	.000	.000	.000		.000	.000	.000	.000	.000
	N	1166	1034	104	1162	168	708	557	426	425	423	467	412	599	360	162	145	482	1179	203	322	342	163	99
Business Studies	Correlation	.609	.601	.537	.602	.600	.629	.550	.646	.612	.625	.670	.658	.558	.607	.579	.500	.566	.452	1	.491	.567	.260	.465
	Sig. (2-tailed)	.000	.000	.000	.000	.000	.000	.000	.000	.000	.000	.000	.000	.000	.000	.000	.000	.000	.000		.000	.000	.026	.001
	N	775	711	71	773	104	444	368	300	298	294	278	254	369	240	104	102	293	203	784	170	214	73	45
Art and Design	Correlation	.551	.520	.439	.483	.447	.422	.449	.463	.471	.448	.505	.575	.491	.524	.405	.473	.525	.363	.491	1	.387	.327	.357
	Sig. (2-tailed)	.000	.000	.000	.000	.000	.000	.000	.000	.000	.000	.000	.000	.000	.000	.000	.000	.000	.000	.000		.000	.000	.000
	N	1607	1436	128	1606	171	908	733	598	590	585	614	551	751	535	238	191	603	322	170	1629	280	180	141
PE	Correlation	.546	.500	.338	.577	.425	.534	.523	.530	.541	.558	.577	.639	.543	.568	.448	.474	.487	.316	.567	.387	1	.383	.514
	Sig. (2-tailed)	.000	.000	.001	.000	.000	.000	.000	.000	.000	.000	.000	.000	.000	.000	.000	.000	.000	.000	.000	.000		.000	.000
	N	1325	1162	96	1320	157	789	620	470	467	467	504	517	619	433	164	152	494	342	214	280	1338	129	94
Drama	Correlation	.563	.549	.621	.423	.133	.439	.428	.368	.321	.354	.410	.558	.531	.401	.400	.538	.336	.428	.260	.327	.383	1	.613
	Sig. (2-tailed)	.000	.000	.000	.000	.335	.000	.000	.000	.000	.000	.000	.000	.000	.000	.000	.000	.000	.000	.026	.000	.000		.000
	N	692	631	73	691	55	421	335	228	221	226	289	195	345	241	85	88	194	163	73	180	129	700	86
Music	Correlation	.526	.535	.674	.536	.548	.468	.505	.399	.402	.443	.483	.517	.555	.489	.440	.556	.335	.415	.465	.357	.514	.613	1
	Sig. (2-tailed)	.000	.000	.000	.000	.000	.000	.000	.000	.000	.000	.000	.000	.000	.000	.000	.000	.000	.000	.001	.000	.000	.000	
	N	562	541	33	567	75	280	243	270	269	269	243	190	273	241	93	78	166	99	45	141	94	86	568

Note: Blank cells denote results which are incomputable due to lack of data (i.e., between subjects which are mutually exclusive in the GCSE syllabus, or those with very small samples).

Table S3: Correlation matrix for subjects included in composites.

		English Language	English Literature	Media Studies	Mathematics	Science Core	Science Additional	Biology	Chemistry	Physics	History	RE	Art and Design	Drama	Music
English Language	Correlation	1	.800	.609	.691	.659	.631	.646	.633	.631	.729	.683	.551	.563	.526
	N	5466	4791	461	5398	2890	2349	2159	2147	2142	2369	2566	1607	692	562
English Literature	Correlation	.800	1	.549	.618	.601	.586	.601	.599	.595	.696	.638	.520	.549	.535
	N	4791	4832	366	4775	2513	2109	2065	2053	2047	2239	2335	1436	631	541
Media Studies	Correlation	.609	.549	1	.508	.498	.520	.539	.429	.420	.591	.434	.439	.621	.674
	N	461	366	467	455	298	239	125	124	124	145	188	128	73	33
Mathematics	Correlation	.691	.618	.508	1	.752	.735	.734	.763	.775	.686	.595	.483	.423	.536
	N	5398	4775	455	5461	2889	2347	2157	2145	2139	2363	2559	1606	691	567
Science Core	Correlation	.659	.601	.498	.752	1	.831				.664	.594	.422	.439	.468
	N	2890	2513	298	2889	2931	2372				1199	1369	908	421	280
Science Additional	Correlation	.631	.586	.520	.735	.831	1				.650	.568	.449	.428	.505
	N	2349	2109	239	2347	2372	2372				1018	1145	733	335	243
Biology	Correlation	.646	.601	.539	.734			1	.828	.821	.678	.620	.463	.368	.399
	N	2159	2065	125	2157			2174	2145	2137	1093	1046	598	228	270
Chemistry	Correlation	.633	.599	.429	.763			.828	1	.834	.681	.609	.471	.321	.402
	N	2147	2053	124	2145			2145	2162	2141	1091	1032	590	221	269
Physics	Correlation	.631	.595	.420	.775			.821	.834	1	.673	.584	.448	.354	.443
	N	2142	2047	124	2139			2137	2141	2157	1081	1035	585	226	269
History	Correlation	.729	.696	.591	.686	.664	.650	.678	.681	.673	1	.689	.505	.410	.483
	N	2369	2239	145	2363	1199	1018	1093	1091	1081	2388	1179	614	289	243
RE	Correlation	.683	.638	.434	.595	.594	.568	.620	.609	.584	.689	1	.491	.531	.555
	N	2566	2335	188	2559	1369	1145	1046	1032	1035	1179	2587	751	345	273
Art and Design	Correlation	.551	.520	.439	.483	.422	.449	.463	.471	.448	.505	.491	1	.327	.357
	N	1607	1436	128	1606	908	733	598	590	585	614	751	1629	180	141
Drama	Correlation	.563	.549	.621	.423	.439	.428	.368	.321	.354	.410	.531	.327	1	.613
	N	692	631	73	691	421	335	228	221	226	289	345	180	700	86
Music	Correlation	.526	.535	.674	.536	.468	.505	.399	.402	.443	.483	.555	.357	.613	1
	N	562	541	33	567	280	243	270	269	269	243	273	141	86	568

Note: This table duplicates relevant correlations from SOM Table 2, for convenience. Mean correlations between the individual subjects included in composites (see SOM Table 1) are as follows:- English: .80, Science: .83, Humanities: .51, Core Subjects: .7. The overall mean correlation (.56) given in the text includes all subjects in the dataset with sufficient sample sizes to analyse (i.e., all subjects listed in SOM Table 2).

Table S4: Sex limitation A, C and E estimates (95% confidence intervals).

	Male			Female		
	A	C	E	A	C	E
Mean grade for GCSE passes	0.57 (0.49 - 0.66)	0.29 (0.20 - 0.37)	0.13 (0.12 - 0.15)	0.47 (0.41 - 0.54)	0.43 (0.36 - 0.49)	0.1 (0.09 - 0.11)
Number of GCSE passes at grade A*-C	0.56 (0.47 - 0.66)	0.26 (0.16 - 0.34)	0.19 (0.17 - 0.21)	0.46 (0.39 - 0.54)	0.38 (0.3 - 0.45)	0.16 (0.15 - 0.18)
GCSE English mean grade	0.54 (0.45 - 0.65)	0.26 (0.16 - 0.35)	0.20 (0.18 - 0.22)	0.48 (0.41 - 0.56)	0.36 (0.28 - 0.43)	0.16 (0.15 - 0.18)
GCSE science mean grade	0.68 (0.58 - 0.80)	0.13 (0.01 - 0.23)	0.19 (0.17 - 0.21)	0.50 (0.42 - 0.59)	0.33 (0.24 - 0.41)	0.17 (0.15 - 0.19)
Mathematics	0.57 (0.47 - 0.68)	0.22 (0.12 - 0.32)	0.21 (0.19 - 0.23)	0.54 (0.46 - 0.63)	0.29 (0.21 - 0.37)	0.17 (0.15 - 0.18)
GCSE core subjects mean grade	0.63 (0.53 - 0.73)	0.23 (0.12 - 0.32)	0.15 (0.13 - 0.16)	0.53 (0.46 - 0.62)	0.35 (0.27 - 0.43)	0.11 (0.10 - 0.12)
GCSE humanities mean grade	0.37 (0.25 - 0.49)	0.35 (0.23 - 0.45)	0.28 (0.25 - 0.32)	0.47 (0.37 - 0.58)	0.29 (0.19 - 0.39)	0.23 (0.21 - 0.26)

Note: All subjects are composites, except for mathematics (the single GCSE). Heritability is consistently higher for males, and shared environment higher for females – except for the humanities composite, for which this pattern is reversed. For females, heritability is similar (around 50%) for all the subjects and composites assessed. For males, it is more variable, reaching 68% for science (the highest heritability found for any subject), and falling almost in half to 37% for humanities (the lowest found for any subject). However, caution is warranted in interpreting these results, for reasons discussed in the text. In addition, the confidence intervals are quite wide, and often overlapping between males and females.

Tables S5-S11

Note: These tables present fit indices for sex-limitation sub-model comparisons. All comparisons are made with the full sex-limitation model. The same pattern of results is shown for all variables: significant quantitative but no qualitative sex differences (however, the null model is the most informative in this instance, as discussed in the text). ep = estimated parameters, Δχ2 = change in chi-square (-2 log-likelihood) between the models, Δdf = change in degrees of freedom.

Table S5: Sex limitation sub-model comparisons: Mean grade for GCSE passes

Model	ep	χ2	df	AIC	Δχ2	Δdf	p
Full sex-limited	9	17292.3	10965	-4637.702	-	-	-
Quantitative differences	6	17352.23	10968	-4583.77	59.93	3	< .01
Qualitative differences (fixed rG)	8	17292.3	10966	-4639.702	0	1	1.00
Qualitative differences (fixed rC)	9	17292.3	10965	-4637.702	0	0	1.00
Null model	5	17352.23	10969	-4585.77	59.93	4	< .01

Table S6: Sex limitation sub-model comparisons: Number of GCSE passes at grade A*-C

Model	ep	χ2	df	AIC	Δχ2	Δdf	p
Full sex-limited	9	18211.53	11050	-3888.469	-	-	-
Quantitative differences	6	18250.71	11053	-3855.294	39.17	3	< .01
Qualitative differences (fixed rG)	8	18211.53	11051	-3890.469	0	1	1.00
Qualitative differences (fixed rC)	9	18211.53	11050	-3888.469	0	0	1.00
Null model	5	18250.71	11054	-3857.294	39.17	4	< .01

Table S7: Sex limitation sub-model comparisons: GCSE English mean grade

Model	ep	χ2	df	AIC	Δχ2	Δdf	p
Full sex-limited	9	18162.12	10882	-3601.883	-	-	-
Quantitative differences	6	18221.53	10885	-3548.472	59.41	3	< .01
Qualitative differences (fixed rG)	8	18162.12	10883	-3603.883	0	1	1.00
Qualitative differences (fixed rC)	9	18162.12	10882	-3601.883	0	0	1.00
Null model	5	18221.53	10886	-3550.472	59.41	4	< .01

Table S8: Sex limitation sub-model comparisons: GCSE science mean grade

Model	ep	χ2	df	AIC	Δχ2	Δdf	p
Full sex-limited	9	17123.15	10124	-3124.847	-	-	-
Quantitative differences	6	17133.86	10127	-3120.136	10.71	3	0.01
Qualitative differences (fixed rG)	8	17123.15	10125	-3126.847	0	1	1.00
Qualitative differences (fixed rC)	9	17123.15	10124	-3124.847	0	0	1.00
Null model	5	17133.86	10128	-3122.136	10.71	4	0.03

Table S9: Sex limitation sub-model comparisons: Mathematics

Model	ep	χ2	df	AIC	Δχ2	Δdf	p
Full sex-limited	9	18118.01	10806	-3493.987	-	-	-
Quantitative differences	6	18130.59	10809	-3487.407	12.58	3	< .01
Qualitative differences (fixed rG)	8	18118.01	10807	-3495.987	0	1	1.00
Qualitative differences (fixed rC)	9	18118.01	10806	-3493.987	0	0	1.00
Null model	5	18130.59	10810	-3489.407	12.58	4	0.01

Table S10: Sex limitation sub-model comparisons: GCSE core subjects mean grade

Model	ep	χ2	df	AIC	Δχ2	Δdf	p
Full sex-limited	9	16224.42	9995	-3765.582	-	-	-
Quantitative differences	6	16252.67	9998	-3743.33	28.25	3	< .01
Qualitative differences (fixed rG)	8	16224.42	9996	-3767.582	0	1	1.00
Qualitative differences (fixed rC)	9	16224.42	9995	-3765.582	0	0	1.00
Null model	5	16252.67	9999	-3745.33	28.25	4	< .01

Table S11: Sex limitation sub-model comparisons: GCSE humanities mean grade

Model	ep	χ2	df	AIC	Δχ2	Δdf	p
Full sex-limited	9	16295.03	9314	-2332.971	-	-	-
Quantitative differences	6	16358.53	9317	-2275.469	63.50	3	< .01
Qualitative differences (fixed rG)	8	16295.03	9315	-2334.971	0	1	1.00
Qualitative differences (fixed rC)	9	16295.03	9314	-2332.971	0	0	1.00
Null model	5	16358.53	9318	-2277.469	63.50	4	< .01
